# From embodying tool to embodying alien limb: sensory-motor modulation of personal and extrapersonal space

**DOI:** 10.1007/s10339-021-01053-2

**Published:** 2021-08-27

**Authors:** Anna Berti

**Affiliations:** Department of Psychology, University of Turin, Via Verdi 10, Turin, Italy

**Keywords:** Near/peripersonal and far/extrapersonal space, Unilateral neglect, Brain damage, Space remapping, Tool use, Pathological embodiment, Multisensory integration, Bimanual coupling

## Abstract

Years ago, it was demonstrated (e.g., Rizzolatti et al. in Handbook of neuropsychology, Elsevier Science, Amsterdam, 2000) that the brain does not encode the space around us in a homogeneous way, but through neural circuits that map the space relative to the distance that objects of interest have from the body. In monkeys, relatively discrete neural systems, characterized by neurons with specific neurophysiological responses, seem to be dedicated either to represent the space that can be reached by the hand (near/peripersonal space) or to the distant space (far/extrapersonal space). It was also shown that the encoding of spaces has dynamic aspects because they can be remapped by the use of tools that trigger different actions (e.g., Iriki et al. 1998). In this latter case, the effect of the tool depends on the modulation of personal space, that is the space of our body. In this paper, I will review and discuss selected research, which demonstrated that also in humans: 1 spaces are encoded in a dynamic way; 2 encoding can be modulated by the use of tool that the system comes to consider as parts of the own body; 3 body representations are not fixed, but they are fragile and subject to change to the point that we can incorporate not only the tools necessary for action, but even limbs belonging to other people. What embodiment of tools and of alien limb tell us about body representations is then briefly discussed.

## Space, tool use and body representation

In everyday life, we act on objects not only in relation to their meaning, but also in relation to the sector of space they occupy. From a purely behavioral point of view, the egocentric space that can be reached by our hand has been defined as near space (or peripersonal space), while the part of space that lies beyond the space that cannot be reached by our hand has been called far space (or extrapersonal space). In this perspective, near and far are used according to linguistic conventions that take our body, or the body of another person, as a reference point to indicate the position of objects in space. One question we can ask is whether ‘near’ and ‘far’ are just linguistic conventions used to denote different points in the environment around us, without implying any brain processing for the position to which the adverbs refer, or whether they instead also have a neural counterpart in different brain representations and in dedicated anatomo-physiological mechanisms. Years ago, many studies in animals showed that different brain areas have a specific neural apparatus to respond to stimuli presented near or far from the animal's body. In particular, it has been found that the representation of far space is primarily served by fronto-parietal oculomotor circuits, in which spatial information is encoded by neurons whose receptive fields are retinocentric, whereas near space is served by separate fronto-parietal networks, in which spatial information is encoded according to egocentric coordinates (e.g., Colby et al. 1996; Leinonen et al. 1979; Colby et al. 1993; Duhamel et al. 1997; see also Rizzolatti et al. 2000 and Berti and Rizzolatti 2002 for a review on this topic). Some of the ‘peripersonal’ neurons are bimodal, i.e., they respond both when a tactile stimulus is presented on the animal's skin and when a visual stimulus is presented in the space adjacent to the receptive skin area (Fogassi et al. 1996; Gentilucci et al. 1988; Graziano and Gross 1995; Graziano et al. 1994). Interestingly, damage to the areas associated with encoding the near space results in a hemineglect in this space, i.e., the monkeys show no response to stimuli presented in the space reachable by the hand or mouth. In contrast, the same stimulus is promptly detected (the animal has an ocular reaction or presents a grasping attempt) if presented in the distant space. The opposite reaction is obtained if the lesion affects the far-space encoding areas (Rizzolatti et al. 1983).

Subsequently, many data showed that also in humans it is possible to observe dissociation between neglect for different sectors of space and even for body space (e.g., Bisiach et al. 1986; Halligan & Marshall 1991; Cowey et al. 1994; Vuillemieur et al. 1998), thus suggesting the existence of discrete neural mechanisms for the representation of spaces analogous to those described in monkeys.

A question the researchers asked at that point was whether 'far' and 'near' are encoded in an absolute way, based on the distance between the body of the subject who is performing the action and the object to which the action is addressed, or whether, on the contrary, the activation of spatial representations can be modulated by factors that modify the spatial relationships between subject and object. A first answer came from a research conducted by Iriki and collaborators (1996) who, studying the properties of bimodal neurons in monkeys, observed that when the animal used a rake to reach for food that was beyond the reach of its hand, the visual receptive fields of those bimodal neurons, anchored to the tactile receptive fields of the arm, were extended to the entire space now reachable with the tool. This finding showed that spatial encoding is not a static and immutable process, but on the contrary has dynamic characteristics that allow the adaptation of space representations to the modification of the spatial relationships between subject and external events. This seminal study is mostly remembered because the main, and until then counterintuitive, discovery was the demonstration that it is possible to remap spaces through the use of tools to the point that a space previously encoded as 'far' is recoded as 'near'. However, this is the first, indirect, evidence that it is possible to incorporate external objects into the representation of the own body. Indeed, Iriki and coworkers interpreted their data by hypothesizing that the rake that made the reaching action possible, was incorporated into the monkey's body schema, probably as an extension of the arm representation. Consequently, peripersonal space was extended up to include what in a static condition was coded as extrapersonal space, thanks to the artificial elongation of a body segment. In one of the first researches conducted on this topic, Berti and Frassinetti (2000) studied a patient who presented a very evident neglect in tests performed in near space, in particular in a bisection test. The bisection test was performed, as always in these cases, by asking the patient to reach with the index finger of her right hand to the midpoint of the line positioned about 40 cm from the subject's body. In the distant space, where the task was, instead, performed—as in Halligan and Marshall's experiment—with a laser pointer, neglect was much less, and in some trials the bisection was completely analogous to that of normal subjects. The patient presented, therefore, a dissociation of awareness between near and far space characterized by the fact that in near space the world to the left of the midline was not taken into account, while events occurring beyond the reach space of the hand were detected in their entirety. This result was analogous to that described earlier by the British authors. Berti and Frassinetti asked, however, that the bisection in far space should not be performed only with the laser, but also using a stick that, unlike the pointer, could reach the line to be bisected. The authors' hypothesis was that if, in the human brain, space representation systems make use of neural mechanisms similar to those described in the primate brain, then the use of the stick should have dynamically altered spatial encoding relationships. Specifically, when distant lines were bisected using the stick, the instrument through which the motor act of reaching was accomplished should have been incorporated into the body schema, extending the patient's arm/hand representation. If near space were also encoded in humans by bimodal neurons that expand the visual receptive field when an instrument is used, then part of the space that before the use of the stick was encoded by the brain as 'near' should, when reached by the instrument, have been recoded as 'far'. Since the patient presented neglect in near space and did not present neglect in far space, the spatial recoding far → near, induced by the use of the tool, should have caused neglect to appear in far space during the reaching motor act. The data collected by Berti and Frassinetti were decidedly in favor of this hypothesis. Indeed, the patient presented a surprising behavior, i.e., the passage, in far space, from spatial awareness (absence of left neglect) when the laser pointer was used, to spatial unawareness (presence of left neglect) when an instrument was used. These data lead the authors to infer a neurophysiological mechanism similar to the one described by Iriki et al. (1996) where the use of a tool, incorporated into the subjects’ body representation, extending the personal space, widened the peripersonal receptive field to include all the space between the subject's body and the stimulus of interest (food, in the case of the monkey; the line in the case of the patient). In a different experimental and pathological context, another surprising case of incorporation in the representation of one's own body of objects of common use was described by Aglioti and collaborators (1996) in a patient who, after a right ischemic injury, presented left hemisomatoagnosia (i.e., she did not recognize as her own the limbs contralateral to the injury). The patient had a ring that when it was put on the right hand was recognized as her own, while when it was removed from the healthy hand and put on the hand affected by the disorder was looked at with amazement and not recognized! Also in this case, it is possible to hypothesize that the ring was incorporated in the representation of the hand to such an extent that if the hand was no longer recognized as part of her body, the ring was not accepted as belonging to her. The evidence summarized so far allows only to infer that the instrument with which the subjects (monkeys or humans) perform the motor act are incorporated in the representation of the body, thanks to the effects observed on the modification of visual receptive fields in monkeys and spatial remapping in patients with neglect. A direct behavioral evidence that the tool is computed within the body representation was presented by Sposito et al. (2012) who showed, in normal subjects, how the use of a stick to complete a reaching/grasping test induced the subjective perception of an elongation of the arm that had used the tool. Indeed, participants were asked before and after a brief training in the use of the tool to indicate the perceived midpoint of the forearm. Participants after the training moved the perceived midpoint distally (as if the arm was longer), thus demonstrating that the tool became part of the arm representation. In different context, other studies show that artificial elongation of the arm by tool use is reflected in kinematic changes so that the kinematic pattern observed after tool-use, was different with respect to pre-training parameters and similar to that of long-armed people (e.g., Cardinali et al. 2009). Overall, these data demonstrate that body representation is willing to accept external objects as parts of one's body if they are critical to performing the task that the subject wants to accomplish. Considering the classical distinction between body schema (‘a highly plastic representation of the body parts, in terms of posture, shape, and size, that can be used to execute or imagine executing movements accurately’) and body image (‘a conscious, lexical, and semantic representation of the body and its parts, with their names and associated functions’) (Martel et al. 2016), the impact of tool use on body representation is considered to affect body schema insofar as its presence modified the perceived metric of the body and has consequences on some motor parameter. In this regard, a crucial point to keep in mind is that both normal subjects and brain-injured patients report no particular sensation of discomfort or abnormal perception of their limbs while using tools that modify arm metrics. In other words, the use of tools (so familiar, convenient, and overlearned in humans) that do not have a shape traceable to body parts have no explicit effect on body awareness and body ownership, that is on body image. The neural bases of the tool incorporation process is, however, far to be clear.

## Embodiment of other’s arm

A well-known disorder described in classical neurology is somatoparaphrenia, where brain-injured patients deny that a limb belongs to their body, often attributing it to other people (REF). Recently, our research group described an opposite symptomatology. Specifically, the delusion that someone else's arm belongs to one's own body. These patients after brain injury show no explicit disownership of the affected arm and, unless put in a particular condition (see below), give correct and consistent responses not only about the structure and identity of bodies in general, but also about their own body parts. Interestingly, they are completely aware of their neurological deficit such as hemiplegia, that is they were not anosognosic. However, if someone else’s arm (for instance the examiner arm) is placed in a body compatible position, internal to the patient’s affected own arm, it is immediately ascribed to the own body and recognized as the own arm. If the owner of the ‘alien’ arm moves it, the patients claim that their own arm has moved, thus becoming anosognosic for the time the ‘alien’ hand is present. We called this clinical entity pathological embodiment (PE), and we indicate the patients with PE as E + patients. Although this phenomenon is similar to the effects of rubber hand illusion (Botvinick and Cohen 1998) in healthy subjects (which consists in attributing to a rubber hand, stimulated simultaneously with one's own hand, the tactile stimuli presented to the real own hand), the main and fundamental difference is that normal subjects in the RHI are aware that the rubber hand does not belong to them, while patients with PE are convinced that the alien hand is theirs. Moreover, pathological embodiment does not occur if, instead of a real hand, a rubber hand is shown (see below). The first question we asked was whether the delusion was a mere verbal confabulation not related to any subjective experience derived from specific neural activity, or whether the patients really experienced the alien arm as their own, thus implying a specific embodied mechanism and some neural counterpart of the delusion of ownership. Following de Vignemont (2011) suggestion according to which ‘ “X” is embodied if some properties of X are processed in the same way as the properties of one’s body’, we searched for evidence that the delusion of ownership influenced patients’ motor and sensory representation and that the alien hand was treated as the own hands. As for the motor side, we reasoned that if patients’ behavior reflects an embodiment mechanism then it should alter patients’ motor representation and, consequently, observable motor parameter (Garbarini et al. 2013). We asked PE patients to perform a bimanual motor task in which they have to draw lines with their healthy hand (usually their right hand) and circles with their affected hand (usually their left hand). This test in healthy subjects determines an ovalization of both the lines and the circles (bimanual coupling effect) because the motor programs of the two hands contaminate each other through the corpus callosum. In our experiment, we used different conditions. In the baseline condition (drawing lines with the right hand and circles with the left hand), patients who were hemiplegic and not anosognosic knew that they could only use their right hand and in this case no ovalization of the lines was observed, because they did not even attempt to move the affected arm. In the crucial condition, in which the alien left hand in an egocentric position drew circles (that is, in the embodiment condition in which the patients believed the alien hand to be their own) the patient's right hand ovalized the lines as if it was influenced by the motor programs of the embodied alien left hand. The ovalization did not occur if the alien hand drew the circles in an allocentric position (i.e., facing the patient), demonstrating that simply observing a hand drawing circles is not sufficient to interfere with motor program of the own hand. The coupling effect found in the embodiment condition clearly implies a deep incorporation of the alien limbs into the patients’ body schema so to affect their motor representations. It further shows that the profoundly altered sense of body ownership of PE patients affects both motor awareness (E + patients, usually aware of their motor impairment, were convinced that their left hand was moving in the embodiment condition) and the sense of agency (E + patients ascribed the alien movements to themselves) (Garbarini et al. 2020). Considering the distinction between body schema and body image, this delusion of ownership seems to affect both body representations. Even more compelling are the data we collected on the sensory consequences of the pathological embodiment. Indeed, we found that pinpricks delivered on the alien embodied hand were felt as if delivered to the real own hand (Pia et al. 2013). Crucially the ‘phantom’ sensation was accompanied by an increase in the skin conductance response (SCR) when the stimuli were delivered on the contralesional alien (embodied) hand (but not on the ipsilesional, not embodied hand) (Garbarini et al. 2014). Going back to de Vignemont’s definition, these behavioral and physiological data clearly show a true ‘embodiment’ of the alien hand into the patients’ body representation insofar as the alien hand receives the same processing as the own hand.

## Physiological embodiment of tool in the pathological embodiment of other’s arm

In the previous paragraph, I presented data that demonstrate the existence of sensory-motor and physiological consequences of the delusion of ownership in PE patients, testifying the embodiment of other people's limbs into patients’ body representation. In the first paragraph, I briefly reviewed data demonstrating how the use of a tool leads to changes in arm metrics without interfering with the feeling of arm ownership. A further question we can ask in PE patients is whether the embodiment of alien arm is so profound and genuine to result in a remapping of representations of space (particularly body space) when the alien (incorporated) hand uses a tool to complete a reaching/grasping task. The observation of such a remapping would further demonstrate that PE, once it is established, acts exactly in the same way as the normal body representation. Therefore, we asked hemiplegic patients with PE to collect different objects on a table using a garbage plier (tool training). In the crucial condition, where embodiment occurred, the alien arm actually performed the task. Before and after the tool training, patients were asked to indicate the midpoint of their own forearm, as in Sposito et al. (2012) paradigm. In the embodiment condition, we expected the patients to be convinced to perform the task with their own arm and, as it happens in tool experiment in normal subjects, to perceive their own arm as longer. Indeed, this was what we found (Garbarini et al. 2015). PE patients, after the tool training (remind that the training was actually performed by the alien hand) indicated the midpoint of the forearm more distally than before the training, showing a significant overestimation of their own arm length. Therefore, PE not only affect the subjective feeling of body ownership affected but it even gates the multisensory integration processes that lead to tool use remapping.

## What tool and alien arm embodiment tell us about body representations

Summarizing the data presented so far, experiments in animals and humans have demonstrated the flexibility of body representations that can incorporate both inanimate objects and body segments of others, following certain constraints. Inanimate object (such as tools, but this can be extended to prosthetic limb, Martel et al. 2016) in order to be embodied must have a fundamental role to accomplish the actions of reaching and/or grasping objects that have specific meaning for the individual. The incorporation, however, in these cases does not go so far as to affect the explicit sense of body ownership. The tool is in fact experienced as an object not belonging to the body, but, coming to be part of the body schema, influences the implicit aspects of body metrics and some kinematic parameters of movement during the execution of the action. The fact that the use of the tool does not affect body ownership, modulating only some implicit metric and motor parameters, without causing discomfort or strange sensations to the acting subject, facilitates the execution of actions, even in brain damaged patients, granting the subject with the best possible representation to carry out the movements. Which areas are involved in this implicit form of embodiment is still to be clarified.

In the pathological conditions of E + patients, another type of embodiment can be observed that instead affects the explicit experience of ownership; the embodiment of other’s arm in brain damaged patients’ body representation. In this form, that seem to affect both body schema and body image, the delusion is characterized by the firm belief that the alien limb belongs to one's own body. To this respect, some points need to be addressed. PE occurs only with *biological hand* and not with rubber hands. This would imply the existence of a pre-existing top down body representations (PEBR) (Tsakiris 2010; Tsakiris and Haggard 2005) for the recognition of a human body in *general.* We called it PEBR-G. (Garbaini et al. 2020), and we proposed that **‘**this representation would store the knowledge about all bodies, it has no specificity with respect to an individual body and, although triggering pictorial and structural memories, it can be considered part of the semantic knowledge. Accordingly, it makes predictions about how human bodies should appear’. This representation is intact in our patients. However, what is striking in these patients is that although able to distinguish between a fake and a real hand they do not visually distinguish between the own and the alien hand. This would suggest a separate pre-existing body representation for the recognition of the *own* body. This representation is affected in our patients. We called it PEBR-O. ‘This representation is more dynamic and continuously updates already existing information about our own body. PEBR-O not only stores the knowledge about our own body but also makes predictions about how a limb should appear and felt if it is our own’ (Garbarini et al. 2020). In other words, ‘it involves predictions related to the shape and visual appearance of the body but also sensory-motor predictions related to body position in space and action’ (IBIDEM) (see also, Schwoebel and Coslett (2005); Romano and Maravita 2014; Romano et al. 2014, 2017). Interestingly, the lesion that characterize PE is a subcortical damage that affects a part of superior longitudinal fasciculus (Pia et al. 2020) that connects areas related to body representations with the pre-motor areas. Notably, pre-motor areas are spared in our patients. We know from the studies on the rubber hand illusion that the feeling of ownership over the fake hand is associated to the activation of the pre-motor cortex (e.g., Ehrsson et al. 2004; Grivaz et al. 2017). Our patients may suggest that in the absence of a control from the PEBR-O on the pre-motor cortex, possibly caused by a disconnection between these two areas due to the brain lesion, the pre-motor cortex tends to incorporate whatever (real) hand is in a position, and has the appearance, compatible to PEBR-G (see Garbarini et al. 2020, for further discussion on this point).

What is to be pointed out is that both in neglect patients and in E + patients who, despite the brain damage, show space remapping by using tool, the neural networks dedicated to tool incorporation must be surely spared in both cases considering that the physiological mechanism to maximize the efficacy of action execution is still possible. Future studies should clarify the neural correlates of tool use incorporation and specify its network and its relation with the subjective body ownership circuit.
